# Supplementary material for: Considerations and recommendations from the ISMRM diffusion study group for preclinical diffusion MRI: Part 1: In vivo small‐animal imaging
Source: Magn Reson Med. 2025 Feb 26;93(6):2507–34. doi: 10.1002/mrm.30429 (PMC11971505; doi:10.1002/mrm.30429)
Supplement: Supplementary file 1 — Data S1: Supplementary Information. [file MRM-93-2507-s001.pdf]

Paper project & timeline

“Consensus on small animal & ex vivo diffusion imaging”

- Goals:
    - Provide the community with a comprehensive set of guidelines or best practices in the field (e.g. a go-to reference for novices or non-specialists)
    - Where a variety of approaches exist, describe their pros and cons or realms of applicability
    - Propose a list of available (publicly shared) code & data specific to our topics, to save everyone the effort of re-inventing the wheel & create the opportunity to improve processing tools and databases as a community
    - Highlight future efforts / challenges in this field
- Manuscript:
    - To be submitted to Magn Reson Med (agreed with editor)
    - Coordinators: Kurt Schilling & Ileana Jelescu (first & last authors of paper)
    - Contributors: author order depending on involvement
- Tentative timeline:
    - Kick-off meeting: summer 2020 – establish task forces on different sections
    - Progress meeting: Nov/Dec 2020
    - Submission: March/April 2021

Summary of survey results

- 24 participants (Thank you!!)
- Results outline:
  1. Added value
  2. Acquisition
  3. Data processing
  4. Perspectives
- Small animal & ex vivo results grouped where similar

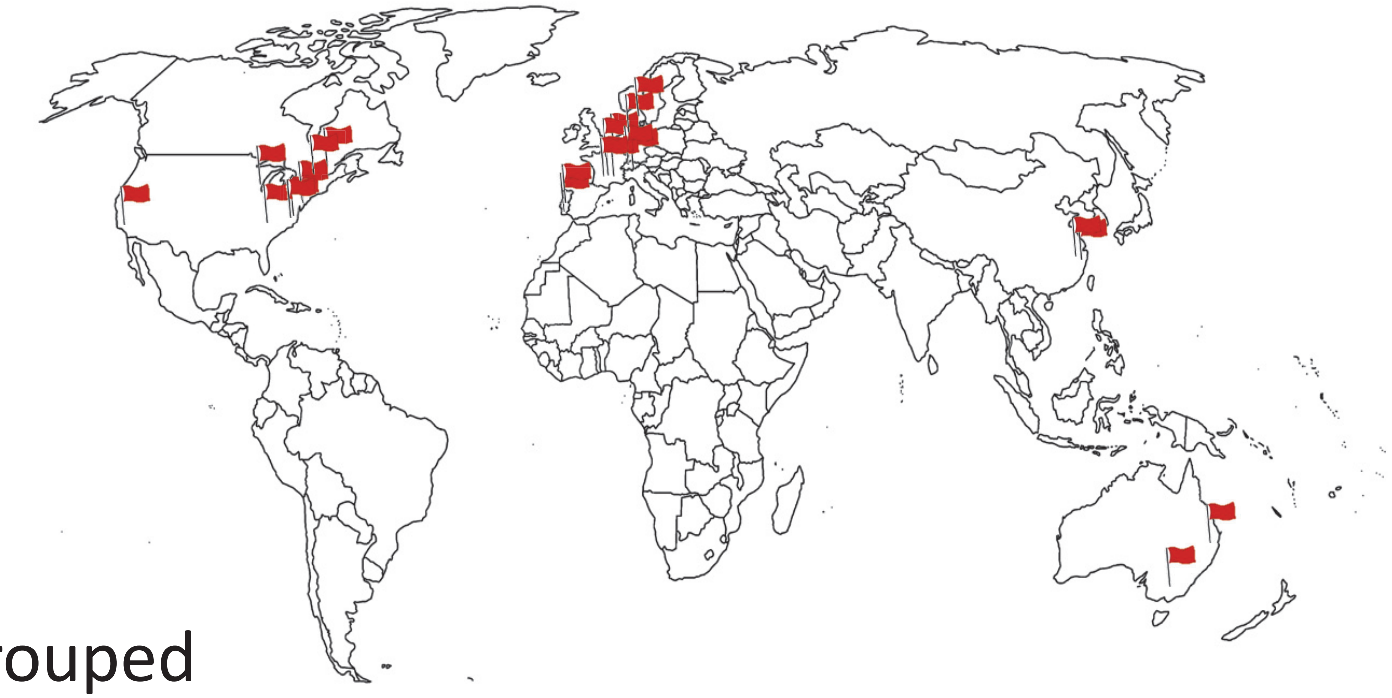

1. Added value

Do you think there is general agreement on the added value of animal imaging to human studies? (i.e., towards validation, invasive procedures, as it relates to scan time, repeated/longitudinal scans)

Added value	Acquisition	Processing	Perspectives

Small Animal	Ex vivo

Added value (from most to least mentioned)

1. Validation, e.g. against histology
2. Diffusion imaging testbed
   - Sequences, model, high SNR, long acquisition
3. Feasibility of longitudinal studies
4. Translational & cross-species studies
5. Disease models & treatments
6. Basic research/biomedical research
7. Connectivity
8. microstructure

Limitations / differences:

1. Scanner/scan differences
   1. Hardware, field, scan time, sequences
2. Variability
   - Diffusion process, animal models
3. Brain structure (WM, cortex, size vs spatial resolution)
4. Consistency of post-processing tools
5. Protocols vary too widely

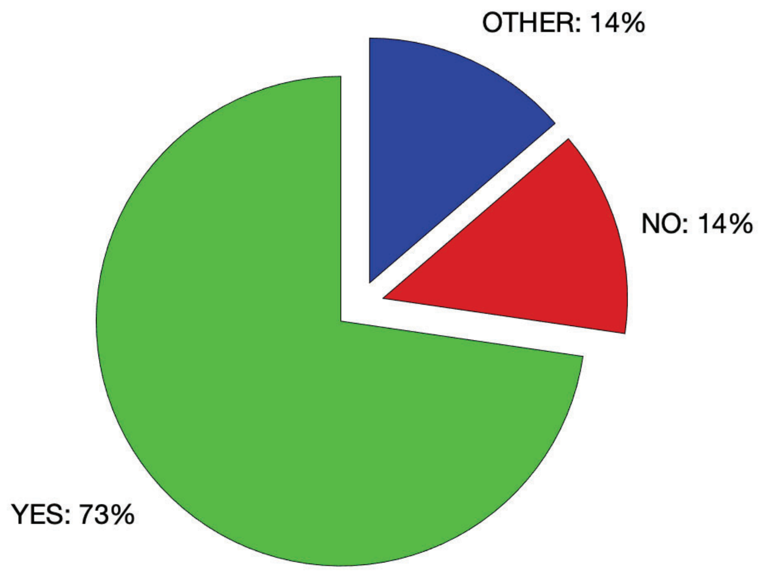

Potential contributors :
Mathieu Santin, Jiangyang Zhang, Luisa Ciobanu, Noam Shemesh, Julien Cohen-Adad, Allan Johnson, Ruiliang Bai, Kurt Schilling, Fatima Nasrallah, Manisha Aggarwal, Yohan van de Looij, Andre Obenaus, Scott Kolbe, Samuel Grant, Denis Le Bihan, Tim Dyrby, Brian Hansen, Ileana Jelescu.

Added value | Acquisition | Processing | Perspectives

Small Animal | Ex vivo

Do you think there is general agreement on the added value of ex vivo imaging to human studies? (e.g. towards validation, spatial resolution, comprehensive acquisition, etc.)

Added value (most to least mentioned)

1. Higher quality scan
   - High SNR, q-t sampling, no motion
2. Allows comparison with histology

Limitations (most to least mentioned)

1. Tissue/diffusion different
   - Diffusivity, fixation, contrast, T1/T2, temperature

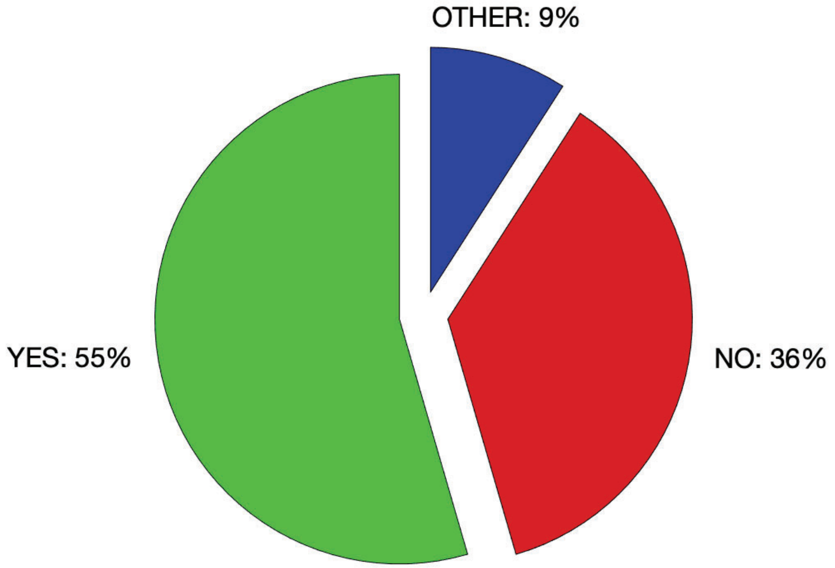

Potential contributors :
Tim Dyrby, Brian Hansen, Mathieu Santin, Jiangyang Zhang, Luisa Ciobanu, Noam Shemesh, Allan Johnson, Ruiliang Bai, Nian Wang, Kurt Schilling, Fatima Nasrallah, Manisha Aggarwal, Yohan van de Looij, Scott Kolbe, Samuel Grant, Ileana Jelescu.

Do you feel that there is agreement on what aspects of small animal imaging are translatable, generalizable to humans, recognized as model of disease/injury for which dMRI is a key or promising biomarker?

Added value	Acquisition	Processing	Perspectives
Small Animal	Ex vivo		

Translatable (most to least mentioned)

1. Brain as model system
    - Neurons, glial cells, microstructure, connectivity, changes, beading, etc.
2. Disease models + mechanisms (TBI, MS)
3. Imaging sequences/principles
4. Identification of "challenges" (crossing fibers)

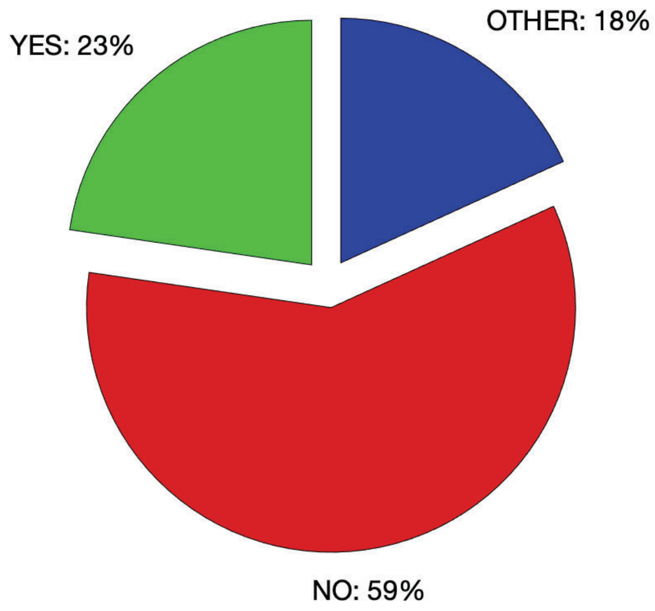

Not Translatable (most to least mentioned)

1. Anatomy (connectivity, complexity, scale differences)
2. Scanning differences (diffusivity, anesthesia, hardware, time)
3. Disease models intrinsic differences

Potential contributors :
Denis Le Bihan, Tim Dyrby, Brian Hansen, Mathieu Santin, Dan Wu, Luisa Ciobanu, Noam Shemesh, Julien Cohen-Adad, Allan Johnson, Kurt Schilling, Fatima Nasrallah, Manisha Aggarwal, Yohan van de Looij, Andre Obenaus, Scott Kolbe, Samuel Grant.

Added value	Acquisition	Processing	Perspectives
	Small Animal	Ex vivo	

Do you feel that there is agreement on what aspects of ex vivo experiments are translatable to in vivo human imaging?

Translatable (most to least mentioned)

1. Tractography + orientation
2. Reproducibility/Robustness/optimization
3. Contrast, models, sequences, encoding and optimization
4. Biomedical questions
5. Microstructure
6. Genetic links

Not Translatable (most to least mentioned)

1. Diffusion time and magnitude (fixation + temperature)
2. Microstructure changes (compartments, exchange, diameters)
3. Acquisition protocols
4. SNR/resolution/acquisition

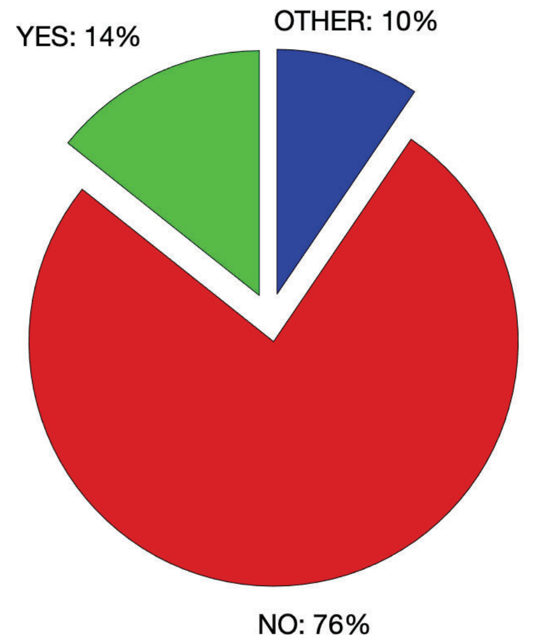

Potential contributors :
Please volunteer! (we forgot to ask ☺)

Added value | Acquisition | Processing | Perspectives

Small Animal | Ex vivo

Are there differences between species that make some models more appropriate for diffusion validation/translation than others?

Rodent models:

1. Down-side: Rodent brain structure v different from human
   - prominent GM, limited WM, opposite of human
   - Lower surface-to-volume area (e.g. TBI)
   - Cell / axon properties (soma size, axon thickness)
2. Up-side: Mouse: easier due to small size, low cost, well characterizes in bred mouse lines, rich resources, large availability
3. Suited when/for:
   - GM strains available that replicate human neurological diseases (stroke, epilepsy…)
   - Body IVIM, non-Gaussian diffusion
   - single fiber structures like corpus callosum

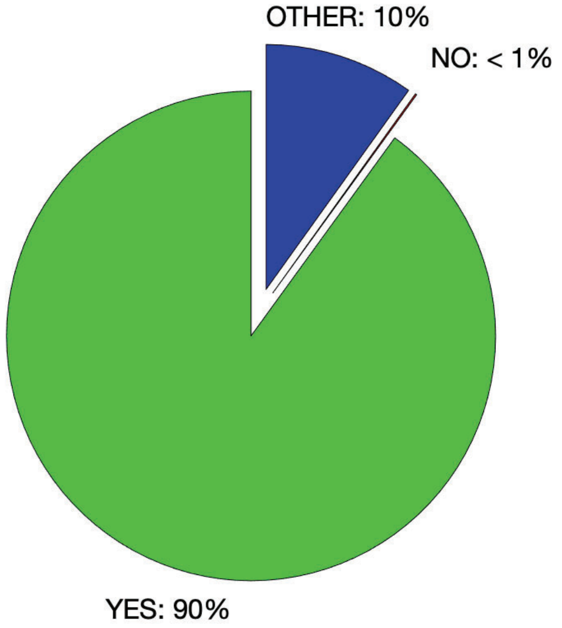

Primate/pig/sheep/marmoset models:

1. Up-side: Closer to human
2. Down-side: Reduced availability, bigger ethical issues
3. Suited for/when:
   - brain development/anatomy, neuro DTI
   - Complex white matter, like human
   - Gyrified cortex

Other models:

1. Aquatic: Cells with different properties than mammalian
2. Marsupials: no corpus callosum

Potential contributors :
Mathieu Santin, Jiangyang Zhang, Julien Cohen-Adad, Fatima Nasrallah, Yohan VDL, Denis Le Bihan, Tim Dyrby, Brian Hansen.

Do you think there is general agreement on considerations for validation (multi-modal) experiments? How in vivo dMRI should be acquired/processed to facilitate comparison with histology, EM, PLI.

Added value | Acquisition | Processing | Perspectives

Small Animal | Ex vivo

Yes (most to least mentioned)

1. Registration
2. Histology quantification
3. Agreement in pitfalls

No (most to least mentioned)

1. Too many measures
   - Diffusion, histology, histology techniques
2. Processing pipelines/tools
3. Acquisition (hardware, protocol variation)
4. When is a measure validated
   - Too qualitative
5. Registration
6. Field evolves too quickly
7. Different brain models (scale, structure)

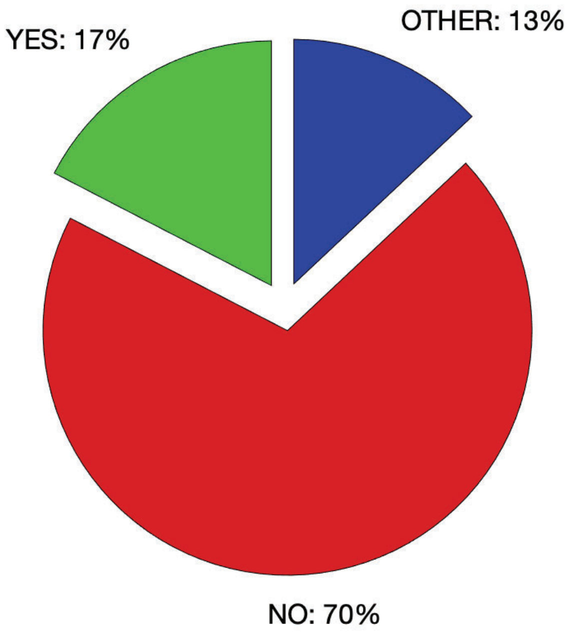

Potential contributors :
Denis Le Bihan, Tim Dyrby, Brian Hansen, Dan Wu, Jiangyang Zhang, Alan Roebroeck, Noam Shemesh, Julien Cohen-Adad, Kurt Schilling, Fatima Nasrallah, Manisha Aggarwal, Andre Obenaus, Samuel Grant.

Added value | Acquisition | Processing | Perspectives

Small Animal | Ex vivo

Do you think there is general agreement on considerations for validation experiments? How ex vivo dMRI should be acquired/processed to facilitate comparison with histology, EM, PLI.

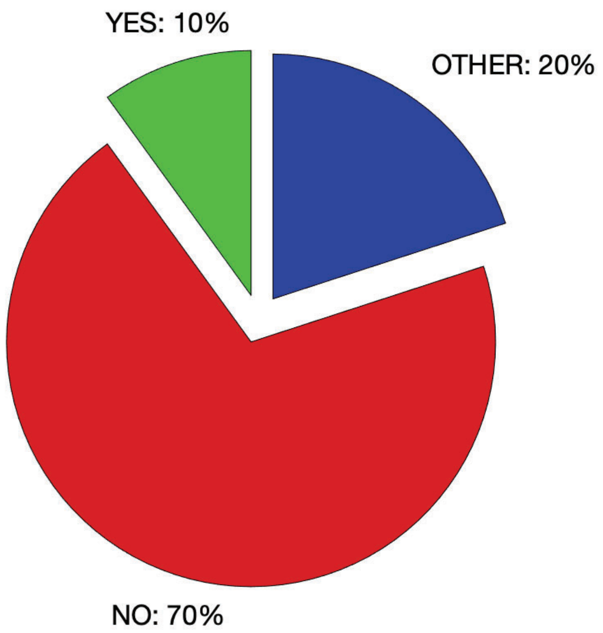

Yes (most to least mentioned)

1. General tissue prep
    - Gd for lower T1, PBS wash out Fix
2. Histology modalities relevant for different features

No (most to least mentioned)

1. Variability in practices/protocols for diffusion
2. Variability in practices/prtoocols for histology prep
3. Quantification of histology measures
4. Dependent on question being answered

Potential contributors :
Tim Dyrby, Brian Hansen, Alard Roebroeck, Noam Shemesh, Allan Johnson, Kurt Schilling, Fatima Nasrallah, Manisha Aggarwal, Samuel Grant.

2. Acquisition

Added value | Acquisition | Processing | Perspectives

Small Animal | Ex vivo

Do you think there are accepted guidelines on diffusion sequences (diffusion encoding + readout)?

Yes (most to least mentioned)

1. SS-EPI (in vivo)
2. 3D EPI (ex vivo)
3. Whatever hardware/vendor allows

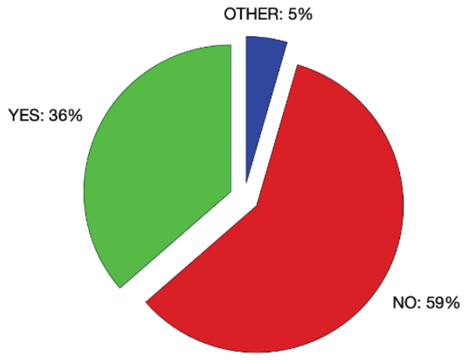

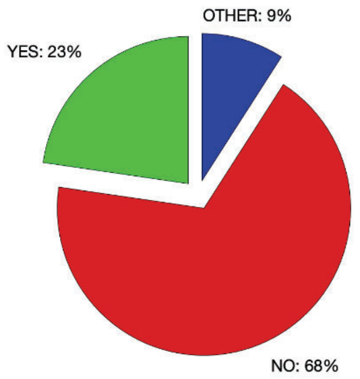

No (most to least mentioned)

1. Dependent on goals
   - Both readout and encoding
2. Gradient capability varies dramatically
3. Too many different readouts/encodings
   - 2d/3d, EPI/RARE/GRASE/STEAM

Potential contributors :
Denis Le Bihan, Tim Dyrby, Brian Hansen, Mathieu Santin, Dan Wu, Jiangyang Zhang, Alard Roebroeck, Noam Shemesh, Julien Cohen-Adad, Allan Johnson, Marleen Verhoye, Andrada Ianus, Ruiliang Bai, Manisha Aggarwal, Samuel Grant, Ileana Jelescu.

Do you think there are accepted guidelines related to acquisition protocol for DTI and higher order models? (as it relates to q-t coverage: b-values, directions, diffusion time)

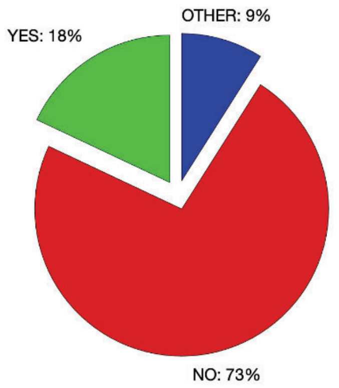

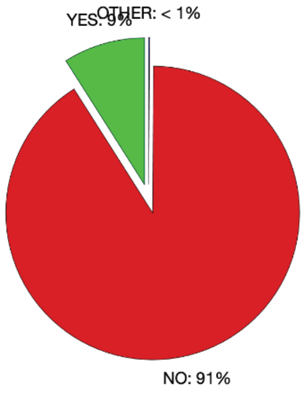

Yes (most to least mentioned)

1. DTI + DKI
2. Reasonable multi-shell recommendations
3. NODDI

No (most to least mentioned)

1. Variability in models
    - Lack of maturity, etc.
2. Dependent on analysis/model
3. Variability in experiment (coils, prep, sequence)

Potential contributors :
Denis Le Bihan, Tim Dyrby, Brian Hansen, Mathieu Santin, Dan Wu, Jiangyang Zhang, Alard Roebroeck, Allan Johnson, Luisa Ciobanu, Noam Shemesh, Marleen Verhoye, Andrada Ianus, Ruiliang Bai, Nian Wang, Fatima Nasrallah, Manisha Aggarwal, Yohan VDL, Samuel Grant, Ileana Jelescu.

Added value | Acquisition | Processing | Perspectives

Small Animal | Ex vivo

Do you think there are accepted guidelines on spatial resolution?
(e.g. range of resolutions, minimum acceptable resolution for given species, 2D vs 3D)

Yes (most to least mentioned)

1. Guidelines welcomed and possible
   - Tractography – aim for isotropic voxels, no slice gap
   - SNR tradeoff
   - Anisotropic voxels slice thickness considerations
   - Brain volume to voxel comparisons

No (most to least mentioned)

1. Dependent on goal/question
2. Dependent on application
3. Dependent on hardware
   - As high as possible given q,b,t limits
4. Wide variation in the literature
   - But can discuss ”what’s” possible

Proposal

1. Give Volume:Volume ratios macaque brain volume = XXXmL, human = XXXXmL, 2mm isotropic human = Xmm isotropic macaque
2. Given CC thickness ratios (for tractography application?)
3. Given axon size ratio (if not 1:1??)

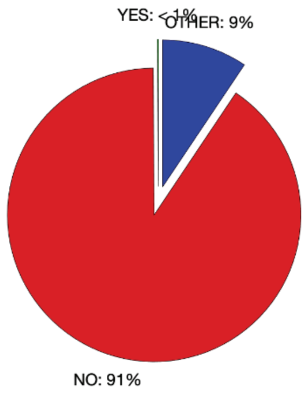

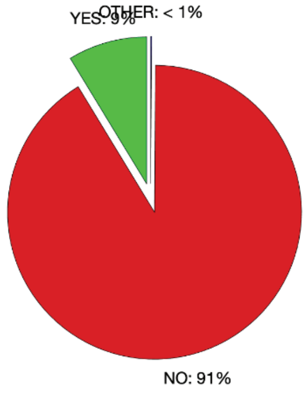

Potential contributors :
Denis Le Bihan, Tim Dyrby, Mathieu Santin, Jiangyang Zhang, Alard Roebroeck, Noam Shemesh, Julien Cohen-Adad, Allan Johnson, Marleen Verhoye, Andrada Ianus, Ruiliang Bai, Nian Wang, Fatima Nasrallah, Manisha Aggarwal, Yohan VDL, Andre Obenaus, Samuel Grant, Ileana Jelescu.

Added value	Acquisition	Processing	Perspectives

Small Animal	Ex vivo

Do you think there are accepted guidelines related to specie- and organ-specific MRI coils? (sizes, types)

Yes (most to least mentioned)

1. Smallest to fit sample (ex vivo)

No (most to least mentioned)

1. Dependent on goal/question
2. Depends on availability
    - Custom coils, different vendors
3. Too much variability

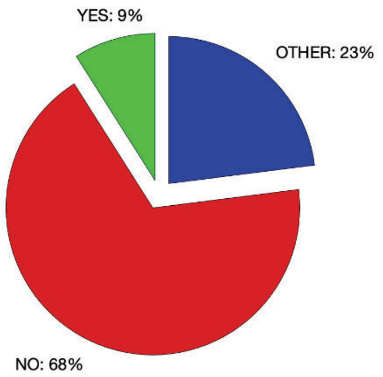

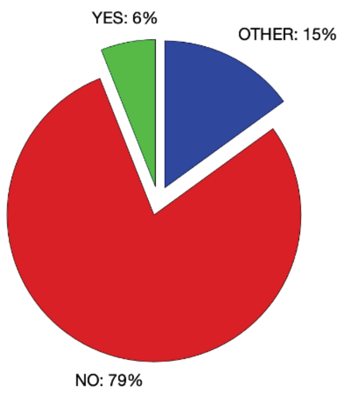

Proposal

1. Discuss (briefly) kinds of coils, effects of size on SNR and homogeneity/FOV
2. Sizes relative to brain (monkey in vivo >XXmm, mouse ex vivo small as XXmm)
3. Pros cons of volume/cryo/custom/quad/linear/single loop

Potential contributors :
Denis Le Bihan, Tim Dyrby, Brian Hansen, Alard Roebroeck, Julien Cohen-Adad, Allan Johnson, Manisha Aggarwal, Fatima Nasrallah, Samuel Grant.

Added value	Acquisition	Processing	Perspectives

Small Animal	Ex vivo

Do you think there are accepted guidelines related to physiological monitoring (anesthesia, target breathing rate, acceptable body temperature fluctuations) and triggered acquisition ?

Yes (most to least mentioned)

1. Need to ”report” them

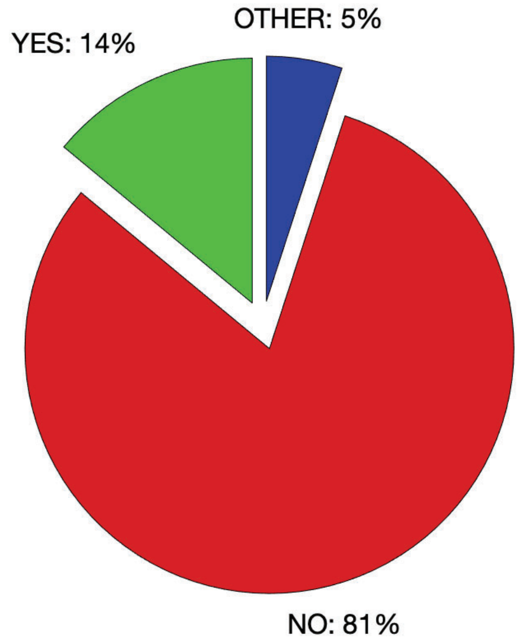

No (most to least mentioned)

1. Effects on diffusion under-studied
    - Vasodilatory/hypoxic effects, temperature
    - Relation to awake
- Not always reported/monitored
- Dependent on resources/environment

Potential contributors :
Tim Dyrby, Brian Hansen, Jiangyang Zhang, Luisa Ciobanu, Noam Shemesh, Marleen Verhoye, Fatima Nasrallah, Manisha Aggarwal, Yohan VDL.

Added value | Acquisition | Processing | Perspectives

Small Animal | Ex vivo

Do you think there are accepted guidelines on ex vivo sample preparation procedures? (including, but not limited to, fixation, rehydration, immersion substance, sample holders)

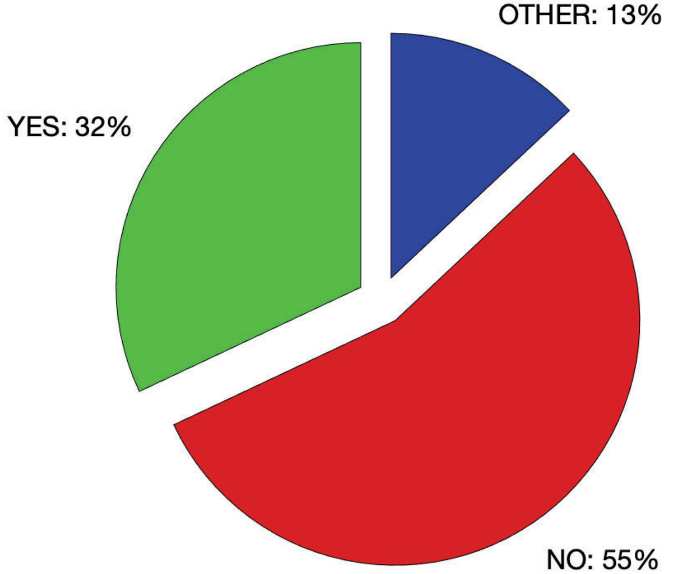

Yes (most to least mentioned)

1. Good practices
   - Rehydrate, image in formalin/PBS/fomblin, temp monitor
- Fix/Gd/Fomblin

No (most to least mentioned)

1. Sample holders
2. Variation in fixatives/length of times/procedure
3. Variation in immersion fluid
4. Need effect of fix/fluid on histology

Prep tips and tricks

1. In skull prep
2. 3d print holders
3. Place in sample ahead of time
4. De-gassing/ vacuum chambers
5. Multiple brains at once

Potential contributors :
Tim Dyrby, Brian Hansen, Mathieu Santin, Jiangyang Zhang, Alard Roebroeck, Noam Shemesh, Allan Johnson, Marleen Verhoye, Andrada Ianus, Ruiliang Bai, Nian Wang, Kurt Schilling, Fatima Nasrallah, Manisha Aggarwal, Yohan VDL, Andre Obenaus, Samuel Grant.

Do you think there are accepted guidelines for ex vivo scan monitoring and scan length? (temperature, tissue degradation, etc.)

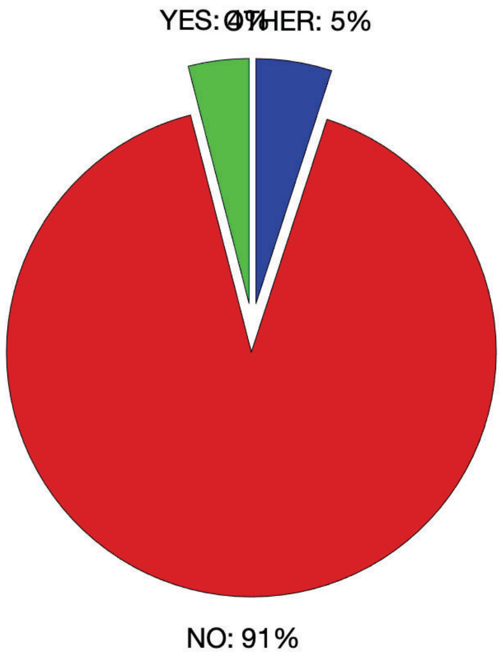

Yes (most to least mentioned)

1. N/A

No (most to least mentioned)

1. Not done/described, little info
2. Temp/stability monitoring important but no guidelines exist

Proposal

1. Propose that monitoring both sample + scanner important
2. Propose how to (and how to fix)

Potential contributors :
Tim Dyrby, Brian Hansen, Mathieu Santin, Jiangyang Zhang, Alard Roebroeck, Manisha Aggarwal.

3. Data processing

Added value | Acquisition | Processing | Perspectives

Small Animal | Ex vivo

Do you think there are accepted guidelines on pre-processing pipelines?

Yes (most to least mentioned)

1. Same as human
2. Adapting of human tools
3. Motion correction, distortion correction, eddy current correction

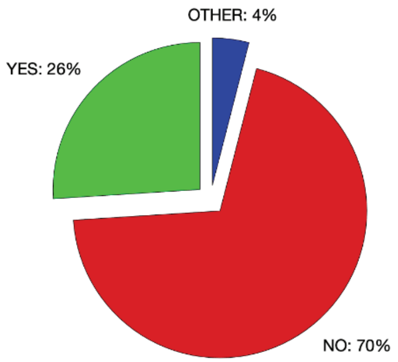

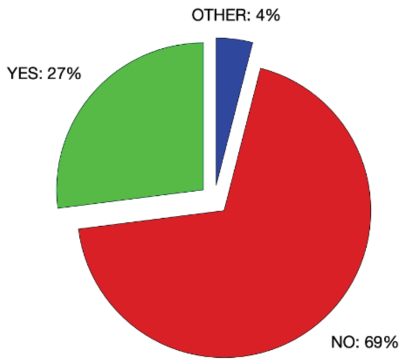

No (most to least mentioned)

1. Human tools semi-adaptable/hackable
2. Many tools available
3. Segmentation, registration, normalization not standardized
4. Dependent upon goals
5. Artifacts and magnitude different (ex vivo)

Tools used and pipelines

1. Mirrors in vivo
   - Exceptions noted: motion correction, denoising?, brain extraction?, Gibbs, susceptibility (if not EPI)

Potential contributors :
Please volunteer! (we forgot to ask ☺)

Added value | Acquisition | Processing | Perspectives

Small Animal | Ex vivo

Please describe resources and tools that you use for processing (software, atlases, etc.)

(most to least mentioned)

1. FSL, Matlab, Mrtrix (tie)
4. In-house scripts
5. SPM
6. DSI Studio
7. DIPY
8. LDDMM
9. ANTs
10. Allen Atlas
11. Many tied
    - AMBC, Waxholm atlas, F99 macaque, JHU Mouse atlas, Study specific templates, dmipy, SCT, qMRILab, NODDI toolbox, DTI-tk

Takeaways

1. Most use FSL for distortion, motion, eddy currents
2. Matlab + in-house likely custom reconstructions
3. MRTrix, DSI Studio common for tractography
4. LDDMM, Ants registration
5. Little disagreement on standard spaces, nothing quite like what we see in human templates/atlases

Do you think there are accepted guidelines for tractography and virtual dissection in small animal and ex vivo imaging? (differences in species, pathways, etc.)

Added value	Acquisition	Processing	Perspectives

Small Animal	Ex vivo

Yes (most to least mentioned)

1. Warping to atlas
2. Rules based on prior knowledge
3. Tools/workflows adapted from human

No (most to least mentioned)

1. Many algorithms
2. Many softwares
3. Atlases not readily available
4. Tools not adaptable to humans
5. Depends on use of tractography/question to answer

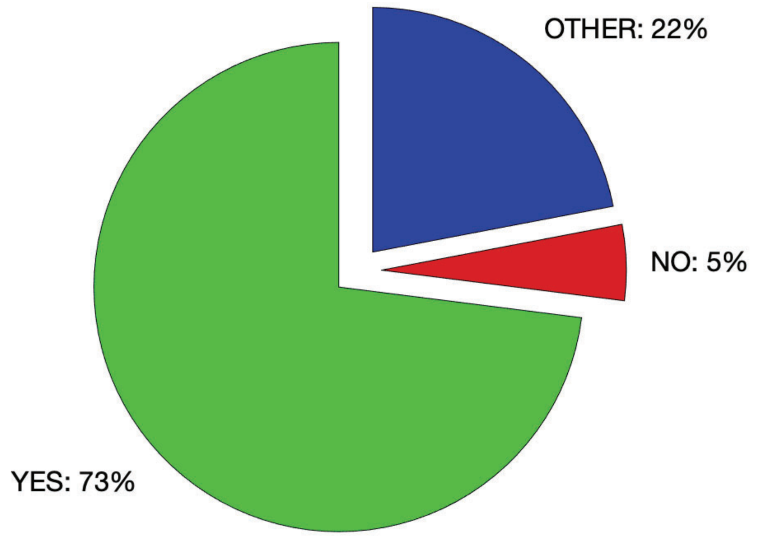

Potential contributors :
Tim Dyrby, Brian Hansen, Alard Roebroeck, Julien CA, Allan Johnson, Kurt Schilling, Manisha Aggarwal, Andre Obenaus.

Added value	Acquisition	Processing	Perspectives

Small Animal	Ex vivo

Do you think there are accepted guidelines for pre-processing related to other organs or spinal cord?

Yes (most to least mentioned)

1. Spinal Cord http://spine-generic.readthedocs.io/
2. Renal: https://pubmed.ncbi.nlm.nih.gov/31676990/

No (most to least mentioned)

1. Still niche applications
2. Brain tools not always translatable/hackable
3. Depends on organ

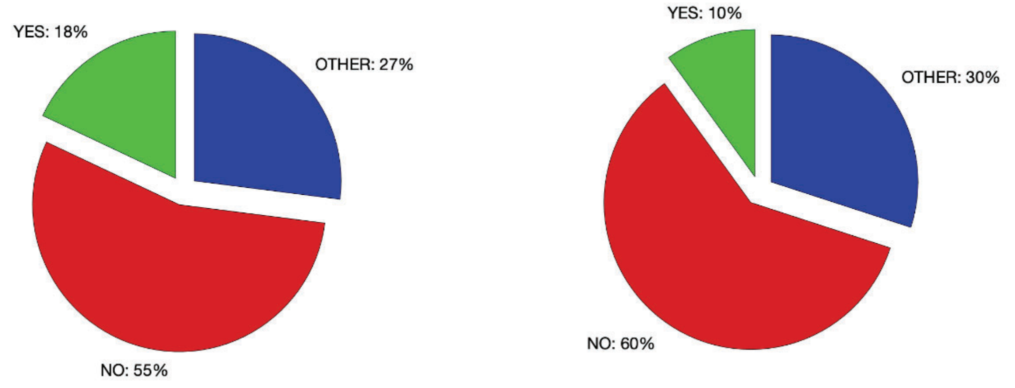

Potential contributors :
Tim Dyrby, Brian Hansen, Mathieu Santin, Alard Roebroeck, Noam Shemesh, Andrada Ianus, Julien CA, Kurt Schilling, Manisha Aggarwal

Added value | Acquisition | Processing | Perspectives

Small Animal | Ex vivo

Do you think there are accepted guidelines for Diffusion analysis?
(changes needed for ex vivo as it relates to relaxation/diffusion properties)

Yes (most to least mentioned)
N/A

No (most to least mentioned)
1. Changes in diffusivity, volume sizes not well understood
2. Leads to necessary (but not studied) changes in parameters, strategies

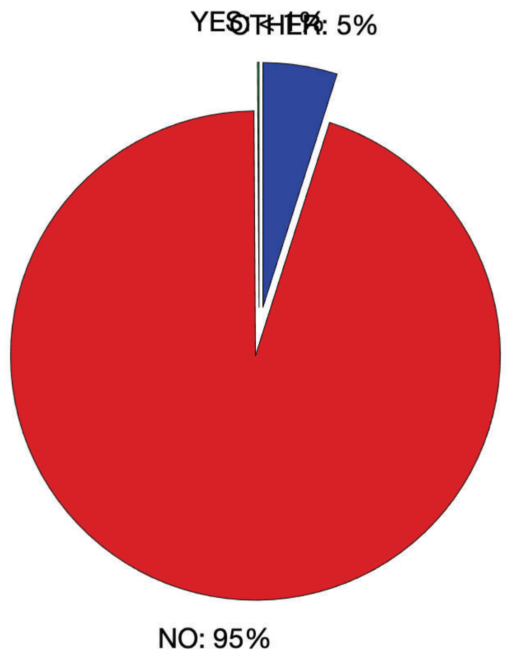

Potential contributors :
Tim Dyrby, Brian Hansen, Mathieu Santin, Jiangyang Zhang, Alard Roebroeck, Noam Shemesh, Andrada Ianus, Ruiliang Bai, Samuel Grant, Ileana Jelescu.

4. Perspectives

Added value | Acquisition | Processing | Perspectives

Small Animal | Ex vivo

Do you have a small animal database, ex vivo database, or histology database, that you are willing to share or have already shared?

Yes

- http://www.drcmr.dk/map-datasets https://osf.io/yp4qg/
- "https://www.nature.com/articles/sdata201672
- https://www.ncbi.nlm.nih.gov/pmc/articles/PMC5024313/
- https://www.sciencedirect.com/science/article/pii/S2352340916304292"
- Our group shared in vivo and ex vivo mouse brain DTI atlases
- mouse brain DTI database and atlas.
- "- White Matter Microscopy Database: https://osf.io/yp4qg/
- - Spine generic multi-subject: https://openneuro.org/datasets/ds001919
- - Spine generic single-subject: https://openneuro.org/datasets/ds002393
- - https://paperpile.com/app/p/bd5ce47f-6546-0ac9-8bfd-8df7ba35472e"
- Sort of : http://www.civm.duhs.duke.edu/SharedData/DataSupplements.htm
- Multi-shell (8-shell, b value up to 8000) ex vivo mouse brain 3D diffusion data at 50 um isotropic resolution
- Validate29 squirrel monkey atlas
- Diffusion MRI atlases of adult (in vivo and ex vivo) and postnatal developing (ex vivo) mouse brains, available at http://cmrm.med.jhmi.edu/
- not shared - but have lots of data
- "Ma Y., Hof P. R., Grant S. C., Blackband S. J., Bennett R., Slatest L., McGuigan M. D., Benveniste H. (2005). A three-dimensional digital atlas database of the adult C57BL/6J mouse brain by magnetic resonance microscopy. Neuroscience 135, 1203–121510.1016/j.neuroscience.2005.07.014
- http://doc.pmod.com/pneuro/pneuro.html?mousebrainatlasma-benveniste-mirrione4996.html"

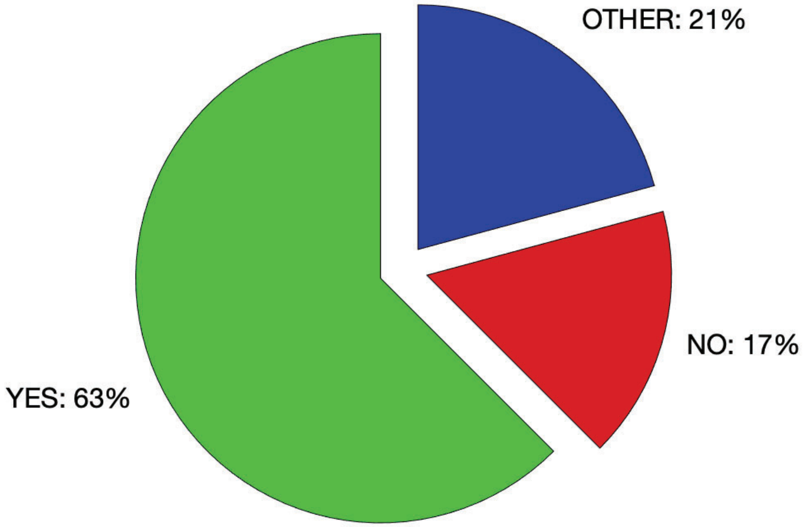

Proposal

Parse through these resources, data? Algorithms? Histology? Invivo/exvivo?

Potential contributors :
Maxime Descoteaux, Tim Dyrby, Brian Hansen, Mathieu Santin, Dan Wu, Jiangyang Zhang, Luisa Ciobanu, Noam Shemesh, Julien CA, Allan Johnson, Nian Wang, Kurt Schilling, Fatima Nasrallah, Manisha Aggarwal, Samuel Grant.

Added value	Acquisition	Processing	Perspectives

Small Animal	Ex vivo

Do you have code for processing pipelines that you are willing to share or have already shared?

Yes (most to least mentioned)

- Tractoflow
- In-house software has been made available to several site worldwide
- "Some DKI tools available here:
- http://cfin.au.dk/cfinmindlab-labsresearch-groups/neurophysics/software"
- "- http://spine-generic.readthedocs.io/
- - https://github.com/sct-pipeline
- - https://github.com/neuropoly/axondeepseg
- - https://github.com/neuropoly/axonpacking"
- DTI qa in general for all analysis (on MASI GitHub)
- http://doc.pmod.com/pneuro/pneuro.html?mousebrainatlasma-benveniste-mirrione4996.html

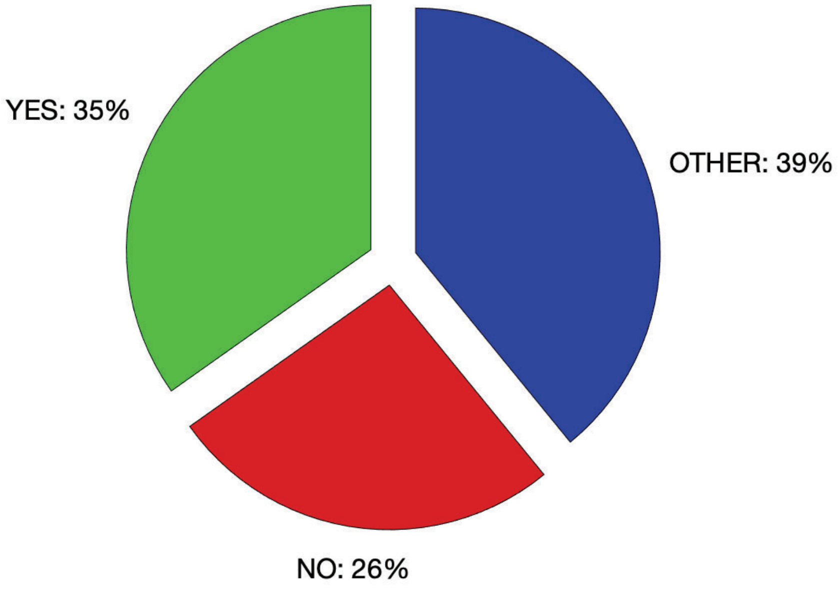

Potential contributors :
Maxime Descoteaux, Brian Hansen, Noam Shemesh, Julien CA, Allan Johnson, Kurt Schilling, Fatima Nasrallah, Samuel Grant.

Added value | Acquisition | Processing | Perspectives

Small Animal | Ex vivo

If you have suggestions to facilitate sharing of code and resources, please indicate them here

Yes (most to least mentioned)

- zenodo or other
- "code: github
- data: OSF, OpenNeuro (although it's been quite buggy for the past couple of months...)"
- through providing full access to data through publications or having a forum for those included in the diffusion group
- My shared pulse sequences: https://osf.io/ngu4a/
- NITRC
- center resources, such as the US National High Magnetic Field Laboratory, for which I am the MRI director could serve as a repository for pre-clinical/ex vivo datasets and processing

What should we strive to achieve as a community as it relates to in vivo small animal diffusion imaging?

Yes (most to least mentioned)

1. Common denominator
    - Acquisition, processing
2. Guidelines for “how to validate”
3. Tools adapted to animals
4. Animal prep guidelines/reporting
5. Improved image quality
6. Data sharing

Potential contributors : all?

Added value | Acquisition | Processing | Perspectives

Small Animal | Ex vivo

What should we strive to achieve as a community as it relates to ex vivo diffusion imaging?

Yes (most to least mentioned)

1. Guidelines for acquisition, processing
2. Tips/common knowledge of differences with in vivo
3. Methods reporting
4. Data sharing
5. Processing pipelines sharing

Potential contributors : all?
